# A signature of immune-related gene pairs (IRGPs) for risk stratification and prognosis of oral cancer patients

**DOI:** 10.1186/s12957-022-02630-1

**Published:** 2022-07-08

**Authors:** Yanling Yu, Jing Tian, Yanni Hou, Xinxin Zhang, Linhua Li, Peifu Cong, Lei Ji, Xuri Wang

**Affiliations:** 1 Department of Stomatology, Weihai Central Hospital, Weihai, China; 2 Department of Stomatology, Feicheng Hospital Affiliated to Shandong First Medical University, Taian, China; 3 Department of Special Dental Care Clinic, Wendeng Stomatology Hospital, Weihai, Shandong China; 4 Repair Department of Stomatology, Shouguang Stomatology Hospital, Weifang, China; 5 Operating room, Weihai Central Hospital, Weihai, China

**Keywords:** Oral cancer, Immunotherapy, Prognosis, Biomarker, Immune infiltration

## Abstract

**Background:**

With low response to present immunotherapy, it is imperative to identify new immune-related biomarkers for more effective immunotherapies for oral cancer.

**Methods:**

RNA profiles for 390 oral cancer patients and 32 normal samples were downloaded from The Cancer Genome Atlas (TCGA) database and differentially expressed genes (DEGs) were analyzed. Immune genesets from ImmPort repository were overlapped with DEGs. After implementing univariate Cox analysis and the least absolute shrinkage and selection operator (LASSO) Cox regression analysis, key immune-related gene pairs (IRGPs) among the overlapped DEGs for predicting the survival risk were obtained. Then, the cutoff of risk score was calculated by the receiver operating characteristic (ROC) curve to stratify oral cancer patients into high and low-risk groups. Multivariate Cox analysis was used to analyze independent prognostic indicators for oral cancer. Besides, infiltration of immune cells, functional annotation, and mutation analysis of IRGPs were conducted. Biological functions correlated with IRGPs were enriched by Gene Set Enrichment Analysis (GSEA) method.

**Results:**

We identified 698 differentially expressed genes (DEGs) in response to oral cancer. 17 IRGPs among the DEGs were identified and integrated into a risk score model. Patients in the high-risk group have a significantly worse prognosis than those in the low-risk group in both training (*P*<0.001) and test (*P*=0.019) cohorts. Meanwhile, the IRGP model was identified as an independent prognostic factor for oral cancer. Different infiltration patterns of immune cells were found between the high- and low-risk groups that more types of T and B cells were enriched in the low-risk group. More immune-related signaling pathways were highly enriched in the low-risk group and Tenascin C (TNC) was the most frequently mutated gene. We have developed a novel 17-IRGPs signature for risk stratification and prognostic prediction of oral cancer.

**Conclusion:**

Our study provides a foundation for improved immunotherapy and prognosis and is beneficial to the individualized management of oral cancer patients.

**Supplementary Information:**

The online version contains supplementary material available at 10.1186/s12957-022-02630-1.

## Introduction

As one of the most common fatal cancer, oral cancer is estimated to lead to 53,260 new cases and 10,750 deaths in 2020 [1]. Several factors have been reported to be the main causes of oral cancer, such as smoking, pan chewing, drinking, and human papillomavirus (HPV) persistent infection [2]. With a high rate of early occurrence and metastasis, the prognosis of oral cancer was poor with a low 5-year survival rate of about 50% [3]. Despite great improvements that have been made for treatments of oral cancer, the survival outcome is still disappointing. Over the past decade, immunotherapy has brought a great breakthrough in cancer treatment.

To strengthen immunotherapy, researchers have focused on the investigation of the tumor microenvironment (TME), which is mainly composed of cytokines and various cells, including immune cells, tumor-associated fibroblasts, and extracellular matrix components [4]. TME plays key roles of various biological behaviors of cancer cells, such as mediating survival, apoptosis, invasion, angiogenesis, and metastasis [5]. Of note, by regulating the crosstalk of tumor cells and stroma, TME is highly involved in immune evasion and suppression of tumor cells, which is critical to tumor initiation, progression, and the response to different cancer therapies [5]. Accumulating evidence has implicated some immune cells and immune-related genes (IRGs) important to the progression of oral cancer, such as myeloid-derived cells, TGFβ, and CCL3 [4, 6–8]. Considering the importance of the immune system in oral cancer, Food and Drug Administration (FDA) has approved some Programmed death-1 (PD-1) inhibitors to treat advanced oral cancer [9]. PD-1 is a receptor expressed on T and B cell surfaces and inhibits the activity of these lymphocytes by binding to its ligands [9]. However, the therapeutic effects were poor with a low response rate of about 20%, from the data that the overall response rate (ORR) of a randomized, open-label, phase 3 trial including 361 recurrent head and neck squamous cell carcinoma (HNSCC) patients receiving nivolumab is 13.3% [10] and ORR of 136 patients with recurrent and/or metastatic HNSCC receiving pembrolizumab is 20% by investigator review [11]. Therefore, to improve the diagnosis and prognosis of patients with oral cancer, identifying new immune-related biomarkers is imperative for more effective immunotherapies.

In the present study, we performed differentially expressed genes (DEGs) and the least absolute shrinkage and selection operator (LASSO) Cox regression analyses to screen out IRG pairs (IRGPs) associated with the prognosis of oral cancer. Then, 17 IRGPs were obtained and integrated into a model for division of risk groups and prognostic prediction of oral cancer patients.

## Materials and methods

### Data collection

Our study was based on the Cancer Genome Atlas (TCGA, https://portal.gdc.cancer.gov/) database. RNA profiles from 390 oral cancer and 32 normal patients were downloaded.

### DEGs in response to oral cancer

DEGs were analyzed by using “edge” package in R. DEGs between oral cancer and normal samples were screened out with the criteria: |log_2_ FC (fold-change)| > 1 and *P* < 0.05.

### Screening for IRGs

Immport database was used to download IRGs and 2,498 IRGs were obtained. The intersection between 2498 IRGs and DEGs was analyzed with a Venn diagram. Subsequently, to overcome technical bias for gene expression, we analyzed the IRGs in pairs. In IRGPs, the ratio between the expression of two IRGs in one patient was set to 1 when the expression level of the latter gene was lower than that of the former. If not, the ratio was set to 0. When the ratios of IRGP were 0 or 1 in more than 80% of the samples, the IRGP was removed. And the remaining IRGPs were selected as candidates.

### LASSO Cox regression analysis of IRGPs

“Caret” package in R was applied to randomly divide the 387 oral cancer samples with survival information into two groups of training and test sets at a ratio of 1:1. Univariate Cox analysis was performed to examine how IRGPs were correlated with the survival of oral cancer. IRGPs significantly associated with the prognosis (*P*<0.01) were used to construct the LASSO Cox regression model. LASSO regression analysis shrinks the subset that reduces the complexity of the model to increase the prediction accuracy and interpretability. We performed 10-fold cross-validation in the LASSO model. Then we calculated the risk score of an individual patient based on the formula as follows: Risk Score= α1×ratioIRGP1+α2×ratioIRGP2+…+αn×ratioIRGPn (α was the coefficient from LASSO analysis, and ratioIRGP was the relative expression of the IRGP). Subsequently, the receiver operating characteristic (ROC) curve was plot using “pROC” package in R to identify the discrimination threshold that distinguishes high-risk patients with low-risk patients. Multivariate Cox analysis was applied to assess the prognostic value of the IRGP model with hazard ratio (HR) and 95% confidence interval (CI).

### Immune cell infiltration in oral cancer

The enrichment of immune cells in the two risk groups was evaluated by using CIBERSORT in R. CIBERSORT estimates the percentage of 22 types of immune cells with the deconvolution method. The radar chart was developed to describe the relative abundance of the immune cells in high- and low-risk groups. When *P* value <0.05, the results were seen as significant.

### Gene set enrichment analysis (GSEA)

GSEA analysis was utilized to analyze the biological function of IRGPs including Gene ontology (GO) and Kyoto Encyclopedia of Genes and Genomes (KEGG) pathway analysis. When P value <0.05, the results were seen as significant.

### Mutation analysis

Mutation analysis was conducted using the IRGs in the model. The “maftoools” package in R was installed to visualize Mutation Annotation Format (MAF), which contained the mutation data in the high-risk group.

## Results

### Identification of IRGs in oral cancer

**Fig. 1:**
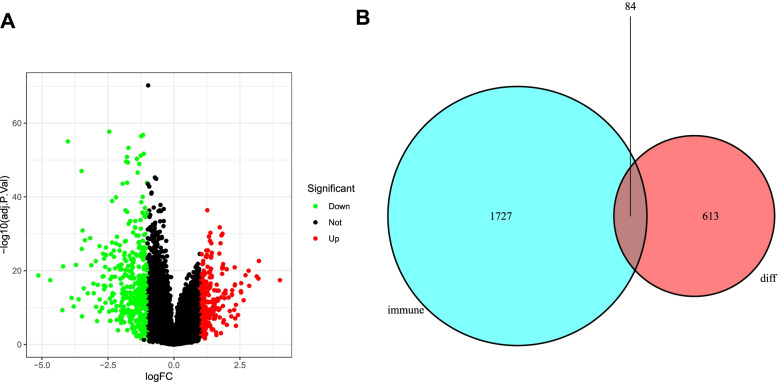
Potential immune-related gene pairs (IRGPs) were identified. **A** Volcano plot of the gene expression in response to oral cancer. The downregulated and upregulated genes were marked by green and red dots, respectively. **B** The shared genes between 698 differentially expressed genes and 2498 IRGs from Immport database were analyzed by Venn diagram

A total of 390 oral cancer and 32 normal samples from the TCGA database were used to identify DEGs. Total 698 DEGs including 257 up-regulated genes and 441 down-regulated genes were identified between oral cancer and normal samples with the criteria |log_2_FC|>1 and *P*<0.05. The distribution of DEGs was visualized by volcano plot (Fig. 1A). Subsequently, 2498 IRGs were downloaded from ImmPort database. And 84 immune-related DEGs were obtained by comparing the 698 DEGs with 2498 IRGs (Fig. 1B). Then to minimize the bias of gene expression from different platforms, the IRGs were analyzed in pairs and 1490 IRGPs were established with the criteria described in Materials and Methods.

### Construction of IRGs model

**Fig. 2:**
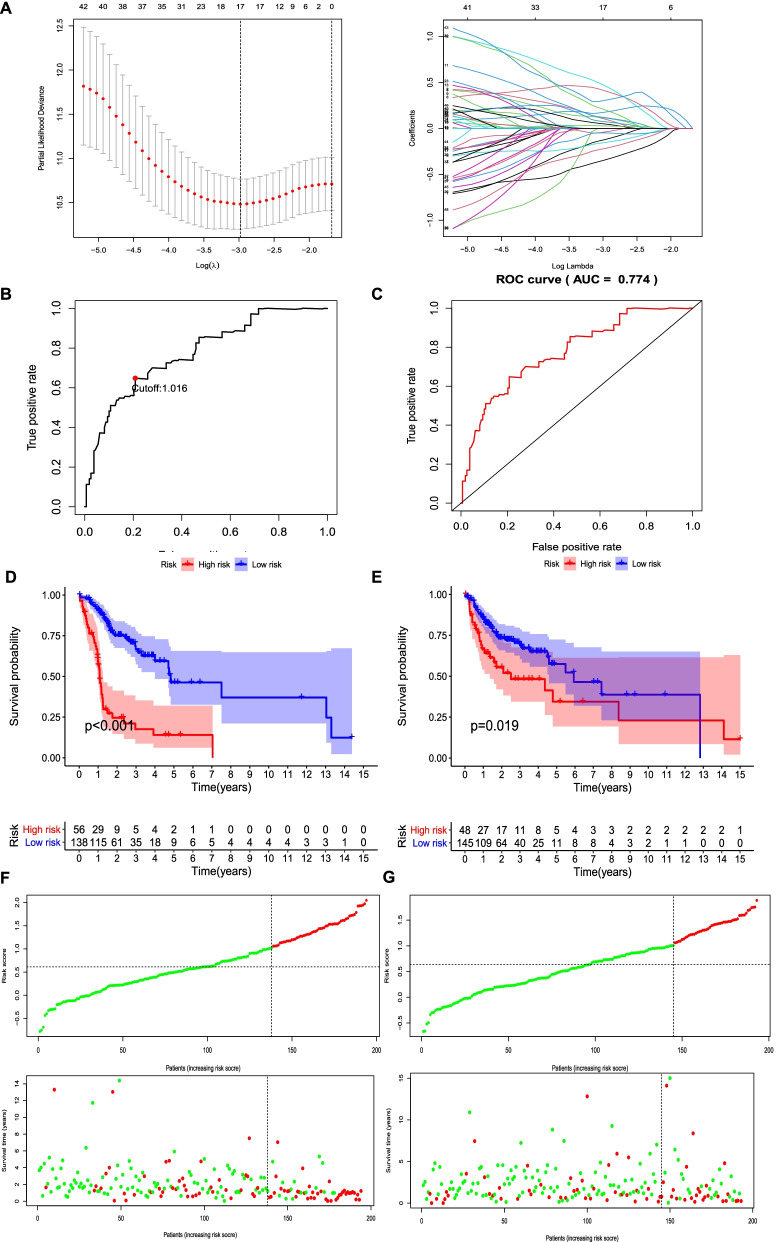
Construction of immune-related gene pairs (IRGPs) model. **A** Prediction accuracy was evaluated by 10-fold cross-validation in the LASSO model. **B** One-year dependent ROC curve was plotted to calculate the optimal cutoff of risk score (red point). **C** Area under the curve (AUC) value for 1-year overall survival rate was calculated in the training set. **D**, **E** High-risk patients had lower overall survival rates than low-risk patients in both training (**D**) and test (**E**) sets. **F**, **G** The number of deaths was increased with the increasing risk score in the training (**F**) and test (**G**) sets. *P*<0.05

The 387 tumor samples with survival information were randomly divided into training (n=194) and test (n=193) sets. After univariate Cox analysis in training set, 49 IRGPs were significantly associated with the prognosis of oral cancer (P<0.01). LASSO Cox regression analysis was performed using the 49 IRGPs and the prediction accuracy was evaluated by 10-fold cross-validation (Fig. 2A). 17 IRGPs were obtained and integrated into the prediction model (Table 1).

**Table 1:** Seventeen immune-related gene pairs were screened out by LASSO regression analysis

Gene	Coefficient
FAM3D|FABP3	− 0.123212435
APOD|PTGDS	0.102940062
APOD|IDO1	0.114162789
APOD|CCL19	0.175640735
PLA2G2A|FABP3	− 0.154866389
PLAU|DEFB1	0.411294017
MMP9|SLURP1	0.118514628
SORT1|IGHG4	0.048939964
ULBP2|IDO1	0.02698727
TYMP|DES	− 0.039797787
OASL|SPP1	− 0.345276271
CLDN4|IGHG2	0.074023063
TNFRSF12A|TNC	0.467337778
RSAD2|TPM2	− 0.049203572
STAT1|IGHG2	0.204728651
TAP1|IGHG2	0.303345321
CXCL11|IL1A	− 0.22016231

Then the risk score (prognostic index) was calculated as the sum of the relative expression of IRGP multiplying its coefficient in the LASSO Cox regression model (Table 1). It was obvious that the coefficients of 6 IRGPs were negative, indicating the relative expression of the IRGPs was negatively associated with the survival of oral cancer patients. Coefficients of the other 11 IRGPs were positive, suggesting a positive correlation. The top 3 IRGPs most strongly and positively correlated with the outcome of oral cancer were TNFRSF12A|TNC, PLAU|DEFB1, and TAP|IGHG2. Significantly, OASL|SPP1 was the most strongly and negatively correlated with the prognosis of oral cancer. One-year dependent ROC curve was plot to stratify the patients into high- and low-risk groups. Figure 2B revealed that the optimal cutoff of risk score was identified as 1.016. The area under the curve (AUC) value for 1-year overall survival rate in the training set was 0.774 (Fig. 2C). With the cutoff in both training and test cohorts, high-risk patients had significantly lower overall survival rates than low-risk patients (Fig. 2D, E; *P*<0.01). And the number of deaths was increased with the increasing risk score in training and test sets (Fig. 2F, G), with the data that in the training group, 73% mortality rate in high-risk patients was much higher than 30% mortality rate in low-risk patients; in the test group, 52% mortality rate in high-risk patients was much higher than 30% mortality rate in low-risk patients.

**Fig. 3:**
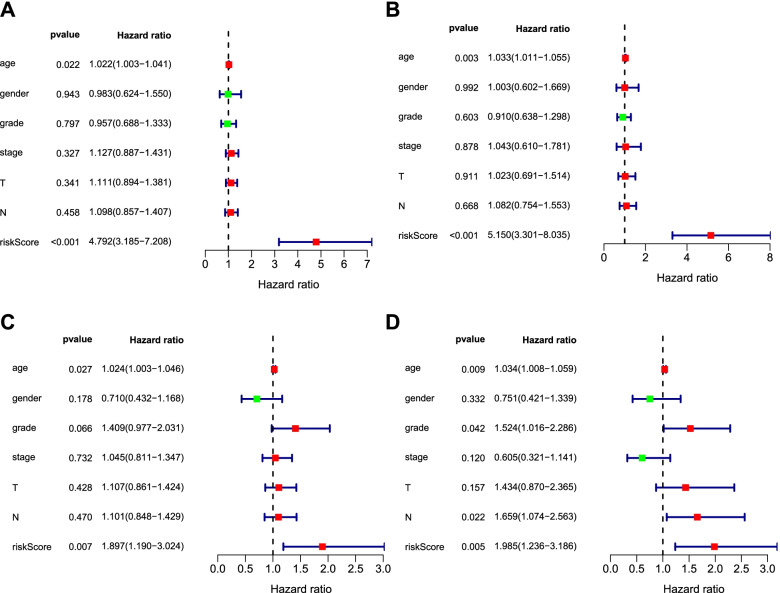
The prognostic value of the IRGP model in oral cancer was examined by univariate and multivariate Cox analyses. **A**, **B** Age and IRGP riskScore were independently associated with survival outcome of oral cancer in the training set. **C**, **D** Age, grade, node stage, and IRGP riskScore could be independent prognostic factors. *P*<0.05

Further, univariate and multivariate Cox analysis was applied to examine the prognostic value of the IRGP model in oral cancer. As shown in Fig. 3A and B, age and IRGP riskScore were independently associated with the survival outcome of oral cancer in the training set (*P*<0.05), in particular, riskScore was more meaningful also from HR and 95% CI (5.150, 3.301–8.035). Age, grade, node stage and IRGP riskScore could be independent prognostic factors (Fig. 3C, D; *P*<0.05) in test set, similarly, riskScore was the most significant indicator also from the values HR and 95% CI (1.985, 1.236–3.186). Therefore, the IRGP model can be seen as an independent factor for oral cancer prognosis.

### Immune cell filtration in high- and low-risk groups

**Fig. 4:**
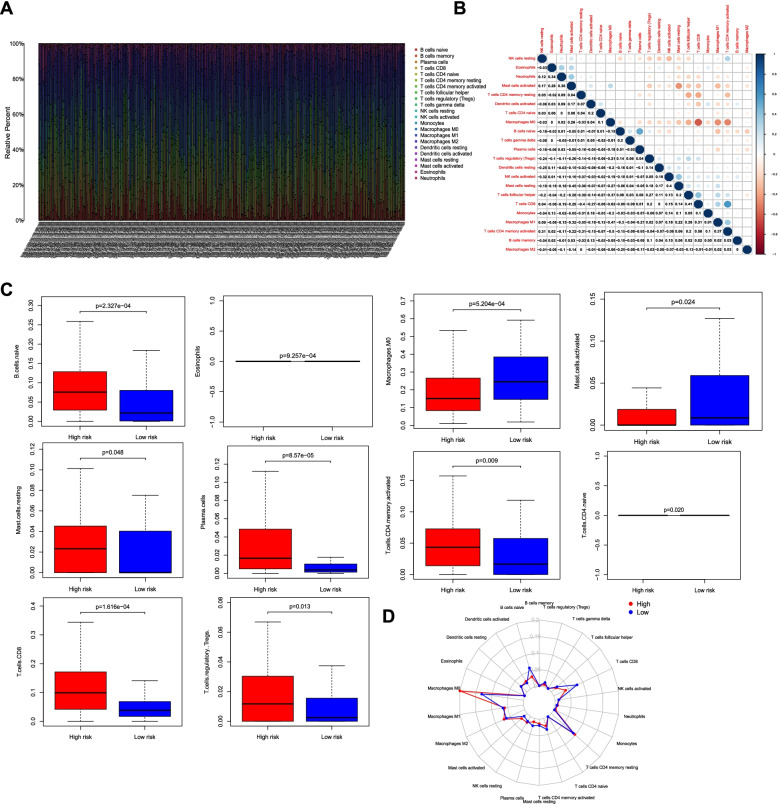
Different infiltration patterns of immune cells were found between high- and low-risk groups. **A** The abundance of 22 types of immune cells varied a lot in different patients. **B** The correlation between the 22 types of immune cells was analyzed between high- and low-risk groups. **C** Comparison of the abundance of the immune cells between the two risk groups. **D** The relative abundance of the 22 types of immune cells in the two risk groups was described by radar chart

It is well accepted that the filtration of lymphocytes into tumors was highly associated with the prognosis of cancer [12]. Next, the association of the 17 IRGPs with infiltrating immune cells in oral cancer was examined with CIBERSORT. Figure 4A displayed that the abundance of 22 types of immune cells varied a lot in different patients. Moreover, we detected the strongest positive correlation between CD8 T cells and activated memory CD4 T cells (Fig. 4B). Naive B cells we also strongly and positively correlated with plasma cells (Fig. 4B). Besides, the strongest negative correlation was found between the CD8 T cells and M0 macrophages (Fig. 4B). M0 macrophages were also negatively correlated with M1 macrophages and activated memory CD4 T cells (Fig. 4B). Among the 22 types of immune cells, 4 types of immune cells including M0 macrophages, activated mast cells, eosinophils and naïve CD4 T cells were highly enriched in the high-risk group in comparison to the low-risk group (Fig. 4C), whereas the low-risk group highly expressed 6 types of immune cells including naïve B cells, resting mast cells, plasma cells, activated memory CD4 T cells, CD8 T cells, and regulatory T cells (Tregs) compared to the high-risk group (Fig. 4C). The radar chart described the relative abundance of the immune cells in the two risk groups (Fig. 4D).

### Functional annotation of the IRGP model

**Fig. 5:**
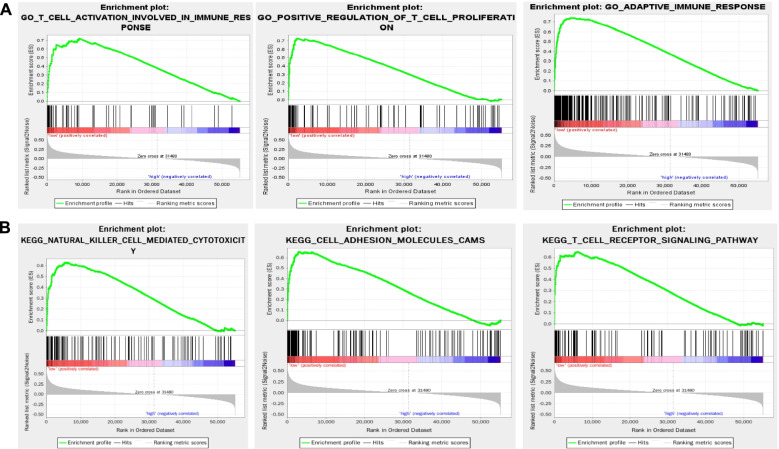
Functional annotation of the IRGP model was analyzed by the GSEA method. **A** Various immune-related GO terms including T cell activation involved in immune response, positive regulation of T cell proliferation, and adaptive immune response were significantly enriched in the low-risk group. **B** Several immune-related signaling pathways including natural killer cell-mediated cytotoxicity, cell adhesion molecules cams, T cell receptor signaling pathway, etc. were significantly enriched in the low-risk group. *P*<0.05

GSEA method was used to discover the biological function of the 17 IRGPs in oral cancer. GO analysis displayed that various immune-related GO terms including T cell activation involved in immune response, positive regulation of T cell proliferation, and adaptive immune response were significantly enriched in the low-risk group (Fig. 5A, Supplementary Table 1). Similarly, KEGG pathway analysis revealed that several immune-related signaling pathways were significantly changed in response to the IRGP model. As shown in Fig. 4B, natural killer cell-mediated cytotoxicity, cell adhesion molecules cams, T cell receptor signaling pathway, etc. were significantly enriched in the low-risk group (Fig. 5B, Supplementary Table 2). These results offered a guide for the investigation of molecular mechanisms by which the 17-IRGPs signature affected the prognosis of oral cancer patients.

### Mutation analysis

**Fig. 6:**
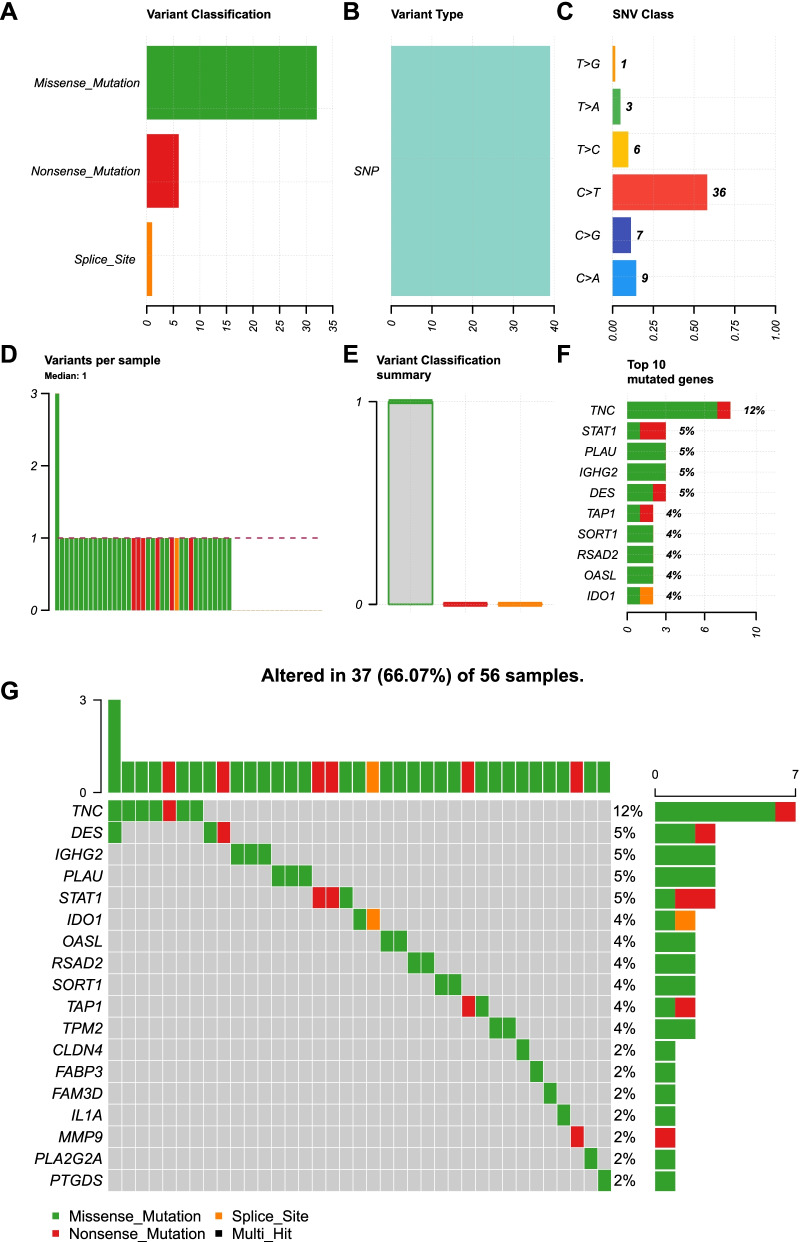
Mutation data of the 17 IRGPs from the high-risk group were analyzed. **A** The mutation types were described by a waterfall plot. **B** The variant type was concentrated entirely on single nucleotide polymorphisms (SNPs). **C** C>T transition represented the largest proportion of SNPs. **D** The maximum number of mutations in each sample was 3 and the median was 1. **E** The number of each variation in each sample was displayed. **F**, **G** Top ten mutated genes were shown

Considering the importance of tumor mutation burden (TMB) in immunotherapy, the mutation data of the 17 IRGPs were analyzed in the high-risk group. The waterfall plot revealed that missense mutation, nonsense mutation, and splice site accounted for the majority of the mutation types (Fig. 6A, E). The variant type was concentrated entirely on single nucleotide polymorphisms (SNPs, Fig. 6B). In terms of SNPs, C>T transition represented the largest proportion (Fig. 6C). The maximum number of mutations in each sample was 3 and the median number was 1 (Fig. 6D). Moreover, Tenascin C (TNC) possessed the largest number of mutations with the highest mutation frequency (Fig. 6F, G).

## Discussion

Oral cancer is a malignant tumor with suppressed immune surveillance. PD-L1 was highly expressed in oral cancer cells and involved in immune escape [13]. In 2017, nivolumab targeting PD-1 was approved to treat advanced oral cancer with metastasis or recurrence [14]. Two years later, pembrolizumab, another PD-1 antibody come into use for oral cancer [15, 16]. However, the therapeutic effects of these PD-1 inhibitors would be mitigated in patients with certain characteristics, such as high expression of inhibitory T-cell receptors [4]. A multicenter cohort has demonstrated the significance of tumor-infiltrating lymphocytes (TILs) using hematoxylin and eosin-stained sections as a potential prognostic marker in early oral tongue cancer, which is introduced by the International Immuno-Oncology Biomarker Working Group and used for standardized determination of TILs in early oral tongue cancer [17]. Thus, it is imperative to identify immune-related markers for precise diagnosis and more individualized immunotherapy of oral cancer. It is hoped that our study will impulse the adoption of immuno-oncology marker for multi-center clinical applications as soon as possible.

To enhance the robustness of prediction, IRGPs were applied to be integrated to the prognostic signature. Herein, gene expression data was not required in this model; instead, the relative expressions of two genes were needed. The application of IRGPs brought about two advantages for prognostic prediction. Firstly, we did not have to perform standardization for gene expression from different platforms. Secondly, the effect of technical bias of different platforms was minimized on gene expression.

At first, 17 IRGPs associated with the prognosis of oral cancer were screened out with DEG and LASSO analyses and integrated into a risk score model. The oral cancer patients were separated into high- and low-risk groups based on the critical risk score point. A higher overall survival rate was detected in low-risk patients. Besides, the mortality risk increased with higher riskScore. Subsequently, univariate and multivariate Cox analyses were performed to assess the correlation between IRGP-model and clinical parameters including age, gender, stage, tumor, and node status. The results revealed that IRGP-model can be considered as an independent prediction factor for oral cancer prognosis. The IRG-based model was widely applied in stratification for various cancers, such as low-grade glioma, ovarian cancer, and melanoma [18–20]. Of note, multiple studies have developed prognostic models for oral cancer frequently based on IRGs. For example, Yao et al. integrated four IRGs and node status to develop a prognostic model for HNSCC [21]. Huang et al. stratified patients with oral squamous cell carcinoma into high- and low-risk groups based on 9 IRGs [22]. However, there is one study based on IRGPs for oral cancer. Zhang et al. constructed a 14-IRGPs signature for HNSCC with a relatively dismal predictive performance (AUC=0.7). The ROC curve of 7 IRGs predicting the 1-year overall survival rate of oral cancer provided the AUC value was 0.660 [23]. Another IRGs signature in oral squamous cell carcinoma predicting the one-year overall survival rate of oral cancer provided the AUC value as 0.753 [24]. These values were lower than our model value as 0.774. In our study, the 17-IRGPs signature had a good predictive performance for oral cancer.

In the signature, the top 3 IRGPs strongest correlated with riskScore were TNFRSF12A|TNC (positive), PLAU|DEFB1 (positive), and OASL|SPP1 (negative). Given the negative correlation between riskScore and prognosis, TNFRSF12A, PLAU, and SPP1 were correlated with prognosis negatively, whereas TNC, DEFB1, and OASL were correlated with prognosis positively. Meanwhile, mutation analysis revealed that TNC was identified as the most frequently mutated gene in high-risk group. Accumulating studies have reported the association of these genes with prognosis in various cancers. Several studies have reported that the high expression of TNFRSF12A and PLAU were involved in a worse outcome for HNSCC patients [21, 25]. Xu et al. found that SPP1 was increased and TNC was decreased in OSCC tissues [26]. The upregulation of SPP1 was involved in a worse outcome for HNSCC patients and the level of TNC expression was decreased with higher stage [26]. However, Chi et al. identified the up-regulation of TNC and OASL in saliva samples from OSCC patients [27]. This is rational that the expression of genes is different in different tissues. Human beta-defensin-1 (DEFB1) has been reported to be a potential tumor suppressor in prostate and renal cancer [28, 29]. Recently, DEFB1 was confirmed to be positively and independently associated with OSCC survival [30]. In addition, the abnormal expression of most other genes has been also reported in various cancers, including oral cancer [31–33]. Collectively, most genes in the model have been investigated in various cancers. In our study, the integration of these 17 gene pairs suggested the important role of the immune system in oral cancer.

Considering the critical role of TME in cancers, we investigated the abundance of infiltrative immune cells in oral cancer. Among the 22 types of immune cells, 10 types were significantly different between high- and low-risk groups. Many studies have explored the prognostic values of immune cells. Song et al. have demonstrated that naïve B cells and regulatory T cells (Tregs) indicated a favorable survival in head and neck cancer [34], whereas eosinophils and activated mast cells indicated a poorer outcome [34]. Besides, eosinophils have been reported to be involved in metastasis and negatively associated with cancer prognosis [34, 35]. Consistently, in our current study, after grouping based on our model, the abundance of naïve B cells and regulatory T cells (Tregs) was significantly enhanced in low-risk patients. And eosinophils and activated mast cells were highly expressed in the high-risk group. Memory T cells are reported to play roles in eliminating tumor cells and activated memory CD4 T cells indicate an improved survival in several cancers [36, 37]. We also identified the high enrichment of activated memory CD4 T cells and CD8 T cells in the low-risk group. In addition, we detected a significant difference of mast cells, plasma cells and naïve CD4 T cells between high- and low-risk groups. Mast cells could influence tumor progression by regulating inflammation [38]. Activated mast cells were found to be associated with a poor prognosis of several cancers [38, 39]. Similarly, in our study, activated mast cells were enriched in the high-risk group, whereas resting mast cells were highly expressed in the low-risk group. The results were consistent with subsequent GSEA analysis that various immune-related GO terms and signaling pathways were enriched in low-risk group, including T cell activation involved in immune response, positive regulation of T cell proliferation, adaptive immune response, natural killer cell mediated cytotoxicity, cell adhesion molecules cams, T cell receptor signaling pathway, etc. These results demonstrated that the immune cells significantly enriched in low-risk group may play promising roles in improving the prognosis of oral cancer, which awaits further investigation.

TMB has been investigated as a promising biomarker in various cancers [40, 41]. SNPs are a common type of gene variation, which are caused by point mutations. SNPs have been reported to be associated with tumorigenesis and prognosis of cancers, including oral cancer [42, 43]. TNC was identified to be the most frequent mutated gene among the 17 IRGPs in oral cancer. There is a possibility that the SNPs affected the immune cells’ infiltration in oral cancer based on a previous study [44]. To our best knowledge, this is the first study that constructed a IRGP-based prognostic model for oral cancer and comprehensively analyzed infiltration of immune cells and TMB. However, there are several limitations in our study. Although the 17-IRGP signature was constructed and validated based on the TCGA database, an individual database should be introduced to validate our model. Our study is retrospective and needs to be corrected for clinical application. Other factors like smoking [45], neoadjuvant chemotherapy [46], heavy alcohol drinking [47], hypoxia [48], aerobic glycolysis [49], and methylation [50] have been discovered to be associated with prognosis of oral cancer, which provides beneficial enlightenments for our future study about IRGPs signature contributing to improve oral cancer outcome.

## Conclusion

In summary, we have developed a novel 17-IRGPs signature for risk stratification and prognostic prediction of oral cancer with excellent predictive performance. High risk score was independently associated with a worse prognosis of oral cancer. Meanwhile, tumors in the low-risk group were infiltrated by more types of immune cells and associated with more immune-related pathways. Further, the model was an independent factor for oral cancer prognosis. Our study provides a foundation for improved immunotherapy and prognosis and is beneficial to the individualized management of oral cancer patients.

## Supplementary Information

**Additional file 1.**

**Additional file 2.**

## Data Availability

The data analyzed in this study are available from the corresponding author on reasonable request.

## Abbreviations

TCGA: The Cancer Genome Atlas
DEGs: Differentially expressed genes
LASSO: Least absolute shrinkage and selection operator
IRGPs: Immune-related gene pairs
ROC: Receiver operating characteristic
GSEA: Gene Set Enrichment Analysis
TNC: Tenascin C
HPV: Human papillomavirus
TME: Tumor microenvironment
IRGs: Immune-related genes
FDA: Food and Drug Administration
PD-1: Programmed death-1
ORR: Overall response rate
HNSCC: Head and neck squamous cell carcinoma
FC: Fold-change
HR: Hazard ratio
CI: Confidence interval
GO: Gene ontology
KEGG: Kyoto Encyclopedia of Genes and Genomes
MAF: Mutation Annotation Format
AUC: Area under the curve
SNP: Single nucleotide polymorphisms
TMB: Tumor mutation burden
TILs: Tumor-infiltrating lymphocytes

## References

[1] Siegel RL, Miller KD: Cancer statistics, 2020. 2020, 70:7-30. 10.3322/caac.21590 31912902

[2] Borse V, Konwar AN, Buragohain P (2020). Oral cancer diagnosis and perspectives in India. Sensors International..

[3] Bao X, Liu F, Lin J, Chen Q, Chen L, Chen F, Wang J, Qiu Y, Shi B, Pan L, et al: Nutritional assessment and prognosis of oral cancer patients: a large-scale prospective study. 2020, 20:146. 10.1186/s12885-020-6604-2 PMC7036168 32087695

[4] Luo JJ, Young CD, Zhou HM, Wang XJ (2018). Mouse Models for Studying Oral Cancer: Impact in the Era of Cancer Immunotherapy. J Dent Res.

[5] Ribeiro Franco PI, Rodrigues AP, de Menezes LB, Pacheco Miguel M (2020). Tumor microenvironment components: Allies of cancer progression. Pathol Res Pract.

[6] da Silva JM (2017). Moreira Dos Santos TP, Sobral LM, Queiroz-Junior CM, Rachid MA, Proudfoot AEI, Garlet GP, Batista AC, Teixeira MM, Leopoldino AM, et al: Relevance of CCL3/CCR5 axis in oral carcinogenesis. Oncotarget.

[7] Lu SL, Reh D, Li AG, Woods J, Corless CL, Kulesz-Martin M, Wang XJ (2004). Overexpression of transforming growth factor beta1 in head and neck epithelia results in inflammation, angiogenesis, and epithelial hyperproliferation. Cancer Res.

[8] Oghumu S, Knobloch TJ, Terrazas C, Varikuti S, Ahn-Jarvis J, Bollinger CE, Iwenofu H, Weghorst CM, Satoskar AR (2016). Deletion of macrophage migration inhibitory factor inhibits murine oral carcinogenesis: Potential role for chronic pro-inflammatory immune mediators. Int J Cancer.

[9] Cohen EEW, Bell RB, Bifulco CB, Burtness B, Gillison ML, Harrington KJ, Le QT, Lee NY, Leidner R, Lewis RL (2019). The Society for Immunotherapy of Cancer consensus statement on immunotherapy for the treatment of squamous cell carcinoma of the head and neck (HNSCC). J Immunother Cancer.

[10] Ferris RL, Blumenschein G, Fayette J, Guigay J, Colevas AD, Licitra L, Harrington K, Kasper S, Vokes EE, Even C (2016). Nivolumab for Recurrent Squamous-Cell Carcinoma of the Head and Neck. N Engl J Med.

[11] Chow LQM, Haddad R, Gupta S, Mahipal A, Mehra R, Tahara M, Berger R, Eder JP, Burtness B, Lee SH (2016). Antitumor Activity of Pembrolizumab in Biomarker-Unselected Patients With Recurrent and/or Metastatic Head and Neck Squamous Cell Carcinoma: Results From the Phase Ib KEYNOTE-012 Expansion Cohort. J Clin Oncol.

[12] Man YG, Stojadinovic A, Mason J, Avital I, Bilchik A, Bruecher B, Protic M, Nissan A, Izadjoo M, Zhang X, Jewett A (2013). Tumor-infiltrating immune cells promoting tumor invasion and metastasis: existing theories. J Cancer.

[13] Badoual C, Hans S, Merillon N, Van Ryswick C, Ravel P, Benhamouda N, Levionnois E, Nizard M, Si-Mohamed A, Besnier N (2013). PD-1-expressing tumor-infiltrating T cells are a favorable prognostic biomarker in HPV-associated head and neck cancer. Cancer Res.

[14] Harrington KJ, Ferris RL, Blumenschein G, Colevas AD, Fayette J, Licitra L, Kasper S, Even C, Vokes EE, Worden F (2017). Nivolumab versus standard, single-agent therapy of investigator's choice in recurrent or metastatic squamous cell carcinoma of the head and neck (CheckMate 141): health-related quality-of-life results from a randomised, phase 3 trial. Lancet Oncol.

[15] Kitamura N, Sento S, Yoshizawa Y, Sasabe E, Kudo Y. Current Trends and Future Prospects of Molecular Targeted Therapy in Head and Neck Squamous Cell Carcinoma. Int J Mol Sci. 2020;22:240. 10.3390/ijms22010240 PMC7795499 33383632

[16] Burtness B, Harrington KJ, Greil R, Soulières D, Tahara M, de Castro G, Jr., Psyrri A, Basté N, Neupane P, Bratland Å (2019). Pembrolizumab alone or with chemotherapy versus cetuximab with chemotherapy for recurrent or metastatic squamous cell carcinoma of the head and neck (KEYNOTE-048): a randomised, open-label, phase 3 study. Lancet.

[17] Heikkinen I, Bello IO, Wahab A, Hagström J, Haglund C, Coletta RD, Nieminen P, Mäkitie AA, Salo T, Leivo I, Almangush A (2019). Assessment of Tumor-infiltrating Lymphocytes Predicts the Behavior of Early-stage Oral Tongue Cancer. Am J Surg Pathol.

[18] Zhang M, Wang X, Chen X, Zhang Q, Hong J (2020). Novel Immune-Related Gene Signature for Risk Stratification and Prognosis of Survival in Lower-Grade Glioma. Front Genet.

[19] Shen S, Wang G, Zhang R, Zhao Y, Yu H, Wei Y, Chen F (2019). Development and validation of an immune gene-set based Prognostic signature in ovarian cancer. EBioMedicine.

[20] Xue YN, Xue YN, Wang ZC, Mo YZ, Wang PY, Tan WQ (2020). A Novel Signature of 23 Immunity-Related Gene Pairs Is Prognostic of Cutaneous Melanoma. Front Immunol.

[21] Yao Y, Yan Z, Lian S, Wei L, Zhou C, Feng D, Zhang Y, Yang J, Li M. Prognostic value of novel immune-related genomic biomarkers identified in head and neck squamous cell carcinoma. J Immunother Cancer. 2020;8:e000444. 10.1136/jitc-2019-000444 PMC7390201 32719094

[22] Huang SN, Li GS, Zhou XG, Chen XY, Yao YX, Zhang XG, Liang Y, Li MX, Chen G, Huang ZG (2020). Identification of an Immune Score-Based Gene Panel with Prognostic Power for Oral Squamous Cell Carcinoma. Med Sci Monit.

[23] Zhao XT, Zhu Y, Zhou JF, Gao YJ, Liu FZ (2021). Development of a novel 7 immune-related genes prognostic model for oral cancer: A study based on TCGA database. Oral Oncol.

[24] Zhu C, Gu L, Yao M, Li J, Fang C (2021). Prognostic Value of an Immune-Related Gene Signature in Oral Squamous Cell Carcinoma. Front Oncol.

[25] Ge Y, Li W, Ni Q, He Y, Chu J, Wei P (2019). Weighted Gene Co-Expression Network Analysis Identifies Hub Genes Associated with Occurrence and Prognosis of Oral Squamous Cell Carcinoma. Med Sci Monit.

[26] Xu G, Wei J, Huangfu B, Gao J, Wang X, Xiao L, Xuan R, Chen Z, Song G (2020). Animal model and bioinformatics analyses suggest the TIMP1/MMP9 axis as a potential biomarker in oral squamous. cell carcinoma.

[27] Chi LM, Hsiao YC, Chien KY, Chen SF, Chuang YN, Lin SY, Wang WS, Chang IY, Yang C, Chu LJ (2020). Assessment of candidate biomarkers in paired saliva and plasma samples from oral cancer patients by targeted mass spectrometry. J Proteomics.

[28] Donald CD, Sun CQ, Lim SD, Macoska J, Cohen C, Amin MB, Young AN, Ganz TA, Marshall FF, Petros JA (2003). Cancer-specific loss of beta-defensin 1 in renal and prostatic carcinomas. Lab Invest.

[29] Sun CQ, Arnold R, Fernandez-Golarz C, Parrish AB, Almekinder T, He J, Ho SM, Svoboda P, Pohl J, Marshall FF, Petros JA (2006). Human beta-defensin-1, a potential chromosome 8p tumor suppressor: control of transcription and induction of apoptosis in renal cell carcinoma. Cancer Res.

[30] Han Q, Wang R, Sun C, Jin X, Liu D, Zhao X, Wang L, Ji N, Li J, Zhou Y (2014). Human beta-defensin-1 suppresses tumor migration and invasion and is an independent predictor for survival of oral squamous cell carcinoma patients. PLoS One.

[31] Attaran N, Gu X, Coates P, Fåhraeus R, Boldrup L, Wilms T, et al. Downregulation of TAP1 in Tumor-Free Tongue Contralateral to Squamous Cell Carcinoma of the Oral Tongue, an Indicator of Better Survival. Int J Mol Sci. 2020;21:6220. 10.3390/ijms21176220 PMC7503265 32867395

[32] Croner RS, Sevim M, Metodiev MV, Jo P, Ghadimi M, Schellerer V, Brunner M, Geppert C, Rau T, Stürzl M (2016). Identification of Predictive Markers for Response to Neoadjuvant Chemoradiation in Rectal Carcinomas by Proteomic Isotope Coded Protein Label (ICPL) Analysis. Int J Mol Sci.

[33] Xin S, Fang W, Li J, Li D, Wang C, Huang Q, et al. Impact of STAT1 polymorphisms on crizotinib-induced hepatotoxicity in ALK-positive non-small cell lung cancer patients. J Cancer Res Clin Oncol. 2021;147:725–37. 10.1007/s00432-020-03476-4 PMC11801888 33387041

[34] Song J, Deng Z, Su J, Yuan D, Liu J, Zhu J (2019). Patterns of Immune Infiltration in HNC and Their Clinical Implications: A Gene Expression-Based Study. Front Oncol.

[35] Nissim Ben Efraim AH (2014). Levi-Schaffer F: Roles of eosinophils in the modulation of angiogenesis. Chem Immunol Allergy.

[36] Novy P, Quigley M, Huang X, Yang Y (2007). CD4 T cells are required for CD8 T cell survival during both primary and memory recall responses. J Immunol.

[37] Liu J, Tan Z, He J, Jin T, Han Y, Hu L, et al. Identification of three molecular subtypes based on immune infiltration in ovarian cancer and its prognostic value. Biosci Rep. 2020;40:BSR20201431. 10.1042/BSR20201431 PMC7593540 33043974

[38] Wang J, Li Z, Gao A, Wen Q, Sun Y (2019). The prognostic landscape of tumor-infiltrating immune cells in cervical cancer. Biomed. Pharmacother..

[39] Cherdantseva TM, Bobrov IP, Avdalyan AM, Klimachev VV, Kazartsev AV, Kryuchkova NG, Klimachev IV, Myadelets MN, Lepilov AV, Lushnikova EL, Molodykh OP (2017). Mast Cells in Renal Cancer: Clinical Morphological Correlations and Prognosis. Bull. Exp. Biol. Med..

[40] Lyu H, Li M, Jiang Z, Liu Z, Wang X (2019). Correlate the TP53 Mutation and the HRAS Mutation with Immune Signatures in Head and Neck Squamous Cell Cancer. Comput Struct Biotechnol J.

[41] Eder T, Hess AK, Konschak R, Stromberger C, Jöhrens K, Fleischer V, Hummel M, Balermpas P, von der Grün J, Linge A (2019). Interference of tumour mutational burden with outcome of patients with head and neck cancer treated with definitive chemoradiation: a multicentre retrospective study of the German Cancer Consortium Radiation Oncology Group. Eur J Cancer.

[42] Deng N, Zhou H, Fan H, Yuan Y (2017). Single nucleotide polymorphisms and cancer susceptibility. Oncotarget.

[43] De Castro TB, Rodrigues-Fleming GH (2020). Gene Polymorphisms Involved in Folate Metabolism and DNA Methylation with the Risk of Head and Neck Cancer. Asian Pac J Cancer Prev.

[44] Jiang AM, Ren MD, Liu N, Gao H, Wang JJ, Zheng XQ, Fu X, Liang X, Ruan ZP, Tian T, Yao Y (2021). Tumor Mutation Burden, Immune Cell Infiltration, and Construction of Immune-Related Genes Prognostic Model in Head and Neck Cancer. Int J Med Sci.

[45] Batta N, Pandey M (2019). Mutational spectrum of tobacco associated oral squamous carcinoma and its therapeutic significance. World J. Surg. Oncol..

[46] Pandey M, Kannepali KK, Dixit R, Kumar M (2018). Effect of neoadjuvant chemotherapy and its correlation with HPV status, EGFR, Her-2-neu, and GADD45 expression in oral squamous cell carcinoma. World J. Surg. Oncol..

[47] Lee DJ, Lee HM, Kim JH, Park IIS, Rho YS (2017). Heavy alcohol drinking downregulates ALDH2 gene expression but heavy smoking up-regulates SOD2 gene expression in head and neck squamous cell carcinoma. World J. Surg. Oncol..

[48] Wu T (2021). Zhang Z-t, Li L, Liu R-y, Bei B-t: Correlation between hypoxia-inducible factor-1α C1772T/G1790A polymorphisms and head and neck cancer risk: a meta-analysis. World J. Surg. Oncol..

[49] Mao L, Wu X, Gong Z, Yu M, Huang Z (2021). PDIA6 contributes to aerobic glycolysis and cancer progression in oral squamous cell carcinoma. World J. Surg. Oncol..

[50] Chen L, Wang D (2021). Identification of potential CpG sites for oral squamous cell carcinoma diagnosis via integrated analysis of DNA methylation and gene expression. World J. Surg. Oncol..

